# OGNNMDA: a computational model for microbe-drug association prediction based on ordered message-passing graph neural networks

**DOI:** 10.3389/fgene.2024.1370013

**Published:** 2024-04-16

**Authors:** Jiabao Zhao, Linai Kuang, An Hu, Qi Zhang, Dinghai Yang, Chunxiang Wang

**Affiliations:** ^1^ School of Computer Science and School of Cyberspace Science, Xiangtan University, Xiangtan, China; ^2^ Hunan Institute of Engineering College of textile and clothing, Xiangtan, China

**Keywords:** graph neural network, ordered message-passing mechanism, microbe-drug association, multi-similarities, prediction model

## Abstract

In recent years, many excellent computational models have emerged in microbe-drug association prediction, but their performance still has room for improvement. This paper proposed the OGNNMDA framework, which applied an ordered message-passing mechanism to distinguish the different neighbor information in each message propagation layer, and it achieved a better embedding ability through deeper network layers. Firstly, the method calculates four similarity matrices based on microbe functional similarity, drug chemical structure similarity, and their respective Gaussian interaction profile kernel similarity. After integrating these similarity matrices, it concatenates the integrated similarity matrix with the known association matrix to obtain the microbe-drug heterogeneous matrix. Secondly, it uses a multi-layer ordered message-passing graph neural network encoder to encode the heterogeneous network and the known association information adjacency matrix, thereby obtaining the final embedding features of the microbe-drugs. Finally, it inputs the embedding features into the bilinear decoder to get the final prediction results. The OGNNMDA method performed comparative experiments, ablation experiments, and case studies on the aBiofilm, MDAD and DrugVirus datasets using 5-fold cross-validation. The experimental results showed that OGNNMDA showed the strongest prediction performance on aBiofilm and MDAD and obtained sub-optimal results on DrugVirus. In addition, the case studies on well-known drugs and microbes also support the effectiveness of the OGNNMDA method. Source codes and data are available at: https://github.com/yyzg/OGNNMDA.

## 1 Introduction

The human microbiome consists of trillions of microbes that reside inside and outside the human body, and these microbes play an essential role in maintaining the overall health of the human body (Ogunrinola et al., 2020). The host-microbe plays a crucial role in several physiological processes in the human body, such as energy collection and storage (Amato et al., 2019), facilitating carbohydrate absorption, and protecting the body from foreign microorganisms and pathogens (Hajiagha et al., 2022). Moreover, the changes in microbiota composition can significantly affect human health Kim et al. (2018); Partula et al. (2019); Catinean et al. (2018). Many studies have shown that the dysbiosis or unbalance of microbiota is closely related to disease, and the microbiota is an important causative factor for many diseases. Therefore, microbes are considered new therapeutic targets for precision medicine (Cullin et al., 2021), and the research on the relationship between microbes and drugs not only aids in drug development but also the diagnosis and treatment of human diseases. However, the popularization and widespread use of antibiotics in modern medicine have led to the emergence of an increasing number of drug-resistant microbes, which seriously threaten human health (Pugazhendhi et al., 2020). Although many researchers have provided extensive evidence on the association between microbes and drugs, traditional biomedical experiments are time-consuming, labor-intensive, and costly (Paul et al., 2010). These reasons hinder the efficiency of drug development and hardly satisfy the massive demands for novel drugs. Therefore, it is necessary to explore the microbe-drug associations at a large-scale level for drug development.

To overcome the above challenges, computational models have emerged as an effective method for identifying microbe-drug associations, and these models are used to predict microbe-drug associations by integrating different genomic information, including genomics, macro genomics, and metabolomics. With the rapid development of high-throughput sequencing technology and advanced genomics techniques, the research on microbe-drug association prediction has developed rapidly, generating a large amount of valuable research data. To further investigate the potential association between microbes and drugs, a series of microbe-drug association databases have been constructed in recent years, such as aBiofilm (Rajput et al., 2018), MDAD (Sun et al., 2018) and DrugVirus (Andersen et al., 2020), which have immensely promoted the development of microbe-drug association prediction models. Over the past few years, many computational models have emerged that utilize the above databases to infer potential associations between microbes and drugs. As an illustration, Zhu et al. proposed a computational method, HMDAKATZ, which applied the KATZ measure to predict inherent associations between microbes and drugs (Zhu et al., 2019b). Long et al. (2020) proposed a computational method called GCNMDA, which combined graph convolutional networks (GCNs) and conditional random fields (CRFs) with an attentional mechanism aiming to identify the hidden associations between microbes and drugs. In 2021, GATMDA was proposed, which utilized inductive matrix completion and graph attention networks (GNNs) to predict associations between microbes and diseases (Long et al., 2021). The Graph2MDA model combined the constructed multimodal attribute graphs and variational graph autoencoder (VGAE) to predict microbe-drug associations accurately (Deng et al., 2022). GSAMDA is likewise a microbe-drug association prediction model, which primarily applies graph attention networks (GATs) and sparse autoencoders (Tan et al., 2022). The computational model NIRBMMDA (Cheng et al., 2022) combines neighborhood-based inference (NI) and restricted Boltzmann machine (RBM) methodologies to predict Microbe-Drug Associations (MDA). By leveraging NI, it extracts proximity information from microbes or drugs, while RBM is used to learn the latent probability distribution inherent in the known association data. This integrative approach harnesses the strengths of both components, resulting in a more robust predictive framework. In the study of Tian et al. (2023), they proposed the SCSMDA model, which was based on GCN and integrated structure-enhanced contrast learning and self-paced negative sampling strategies to improve the accuracy in microbe-drug association prediction. In addition, the GACNNMDA model integrated a GTA-based autoencoder and a CNN-based classifier, which transforms multiple attribute combinations of the microbes and drugs into two feature matrices to predict the associations of the microbes and drugs (Ma et al., 2023). Qu et al. (2023) proposed MHBVDA to predicts virus-drug associations by integrating multiple biological data sources and employing integrating two matrix decomposition-based methods. And it innovatively applies Bounded Nuclear Norm Regularization (BNNR) with regularization terms to mitigate the impact of noisy data and overfitting issues, thereby enhancing prediction accuracy. However, these methods based on graph neural networks still have room for improvement in prediction performance. When multi-layer networks are stacked, there is some confusion between different orders of neighborhood information, the node representations become indistinguishable, and the network performance decreases, which tends to prevent GNN with multiple layers from effectively utilizing the higher-order neighborhood information (Li et al., 2018).

Therefore, to achieve better prediction performance, inspired by the work of Song et al. (2023), this paper proposed an ordered gating mechanism-based ordered message-passing GNN method to infer potential microbe-drug associations, called OGNNMDA. In OGNNMDA, the known association data are preprocessed to compute Gaussian interaction profile kernel similarity and additional biomedical information similarity (microbe functional similarity, drug structural similarity) for drugs and microbes, respectively. Then, the multiple similarity matrices are fused and stitched together to obtain the heterogeneous networks. The heterogeneous network was fed into the encoder consisting of the two-layer fully connected network and the 12-layer ordered message-passing GNN to derive embedding representations of the drugs and microbes, respectively. Finally, the bilinear decoder was adopted to reconstruct the microbe-drug association matrix to infer possible associations between the microbes and drugs. Furthermore, to evaluate the predictive performance of OGNNMDA, in-depth comparative experiments, ablation experiments, and case studies are conducted in this paper. The results demonstrate that OGNNMDA outperforms current representative existing methods and achieves satisfactory results in potential drug-microbe association prediction.

## 2 Datasets

All the aBiofilm, MDAD and DrugVirus datasets provide important insights into the complex interactions between the drugs and the microbes, providing researchers in the fields of bioinformatics and graphical neural networks with a wealth of information to analyze and utilize to advance their studies and methods. The basic statistical information of the three datasets is presented in Table 1.

**TABLE 1:** Statistical information about the datasets.

Dataset	Drugs	Microbes	Associations
aBiofilm	1740	140	2,884
MDAD	1,373	173	2,470
DrugVirus	175	95	933

### 2.1 aBiofilm

In 2018, Rajput et al. introduced the aBiofilm (http://bioinfo.imtech.res.in/manojk/abiofilm/) dataset, which is of great significance for the development of the bioinformatics and graph neural network fields (Rajput et al., 2018). Over the last three decades, many anti-biofilm agents have been experimentally verified to disrupt biofilms. aBiofilm organizes these data, which contain a database, a predictor, and a data visualization module. The database contains biological, chemical, and structural details of 5,027 anti-biofilm agents (1720 different ones) reported from 1988 to 2017. After eliminating redundant associations among them, a total of 2,884 known interaction associations of 1720 drugs and 140 microbes were finally obtained.

### 2.2 MDAD

MDAD (https://github.com/Sun-Yazhou/MDAD/) is also a valuable microbe-drug association dataset, which was proposed by Sun et al. based on a variety of drug-related databases as well as a large amount of literature (Sun et al., 2018). Specifically, MDAD contains 5,505 associations between 180 microbes and 1,388 drugs collected from 993 documentation. After filtering out redundant information, a total of 2,470 microbe-drug associations were obtained, involving 173 microbes and 1,373 drugs.

### 2.3 DrugVirus

DrugVirus (https://drugvirus.info/) compiles interactions involving 118 virus-targeting drugs and 83 human viruses, encompassing SARS-CoV-2 (2019-nCoV) (Andersen et al., 2020). Building upon this foundation, Lond et al. systematically extracted and curated 57 drug-virus associations from pertinent drug databases and scholarly publications, which involved 76 unique drugs and 12 distinct viruses. Ultimately, they assembled a dataset comprising 175 drugs and 95 viruses, yielding a total of 933 documented drug-virus interaction records.

## 3 Preprocessing

In this section, firstly, the definition of the association adjacency matrix is given, secondly, the similarity calculation of drugs and microbe based on the adjacency matrix is given, and finally, the heterogeneous network is obtained based on multiple similarities.

For simplicity, for each dataset, let $D=\left\{d_{1},d_{2},\ldots ,d_{N_{d}}\right\}$ denote the set of different drugs, and $M=\left\{m_{1},m_{2},\ldots ,m_{N_{m}}\right\}$ denote the set of different microbes. Therefore, a primitive known microbe-drug association network $Net=\left\{D\cup M,E\right\}$ can be constructed: for each given drug $d_{i}\left(1\leq i\leq N_{d}\right)$ and microbe $m_{j}\left(1\leq j\leq N_{m}\right)$ there exists a unique edge corresponding to it in *E* if and only if there is a known association between them. Based on the above definition, the adjacency matrix $A\in R^{N_{d}\times N_{m}}$ can be obtained as shown in Eq. 1.

$$A_{i,j}=\left\{\begin{array}{cc} 1\; & \text{if drug }d_{i}\text{ and microbe }m_{j}\text{ has interaction association},\\ 0\; & \text{otherwise} \end{array}\right.$$ (1)

That is, for any given $d_{i}\left(1\leq i\leq N_{d}\right)$ and $m_{j}\left(1\leq j\leq N_{m}\right)$ , there is *A*_*i*,*j*_ = 1 if and only if there is an edge between them in *E*. Otherwise, *A*_*i*,*j*_ = 0.

### 3.1 Constructing drug-drug similarity networks

First, considering that the functions of drugs are determined by their microstructures, and drugs with similar structures have similar chemical properties. So, the SIMCOMP2 tool based on the maximum common substructure between drugs is used in this paper to calculate the drug structure similarity (Hattori et al., 2010). For two drugs *d*_*i*_ and *d*_*j*_ respectively, their structure-based similarity can be expressed as *DSS*(*d*_*i*_, *d*_*j*_). After calculating all the similarities between all drug pairs, an *N*_*d*_ × *N*_*d*_ matrix $DSS\in R^{N_{d}\times N_{d}}$ can be obtained to represent the chemical structure similarities between *N*_*d*_ different drugs.

Next, for any two given drugs or microbes, the Gaussian interaction profile kernel similarity between them is calculated herein by utilizing a Gaussian kernel function based on known microbe disease associations as shown in Eq. 2:

$$DGS\left(d_{i},d_{j}\right)=\exp\left(-\gamma_{d}{\left\|A\left(i,:\right)-A\left(j,:\right)\right\|}^{2}\right)$$ (2)

where *A* (*i*, :) and *A* (*j*, :) denote the *ith* and *jth* rows of the adjacency matrix *A*, respectively, and *γ*_*d*_ denotes the drug-normalized kernel bandwidth, which can be calculated by Eq. 3.

$$\gamma_{d}=\frac{1}{\frac{1}{N_{d}}\sum_{i=1}^{N_{d}}\left({\left\|A\left(i,:\right)\right\|}^{2}\right)}$$ (3)

### 3.2 Constructing microbe-microbe similarity networks

Also, this paper measures microbe similarity in two ways. The first one is the functional similarity of microbe proposed by Kamneva (2017). This computational method is mainly based on the microbial gene family information kernel protein-protein interaction association network. The second similarity between microbes is the Gaussian interaction profile kernel similarity MGS. similar to the drug similarity based on the Gaussian interaction profile kernel, for any given microbe pair *m*_*i*_ and *m*_*j*_, it is computed using the Gaussian kernel function based on the known microbe drug associations as shown in Eq. 4.

$$MGS\left(m_{i},m_{j}\right)=\exp\left(-\gamma_{m}{\left\|A\left(:,i\right)-A\left(:,j\right)\right\|}^{2}\right)$$ (4)

where *A* (:, *i*) and *A* (:, *j*) denote the *i*th and *j*th columns of the adjacency matrix *A*, respectively, and *γ*_*m*_ denotes the microbe normalized kernel bandwidth that can be computed according to Eq. 5.

$$\gamma_{m}=\frac{1}{\frac{1}{N_{m}}\sum_{i=1}^{N_{m}}\left({\left\|A\left(:,i\right)\right\|}^{2}\right)}$$ (5)

### 3.3 Constructing the heterogeneous network

Considering that not all drugs have their structures retrieved from databases, it is not possible to obtain all chemical structure similarities between drugs lacking structural information and other drugs. Therefore, in this paper, a comprehensive similarity is constructed to estimate the similarity between drugs and microbes by integrating Gaussian interaction profile nuclear similarity, microbe functional similarity, and drug chemical structure similarity. Specifically, for any two given drugs *d*_*i*_ and *d*_*j*_, the integrated similarity between them is calculated as shown in Eq. 6:

$$DS\left(d_{i},d_{j}\right)=\left\{\begin{array}{cc} \frac{1}{2}\left(DSS\left(d_{i},d_{j}\right)+DGS\left(d_{i},d_{j}\right)\right) & \text{if }\;DSS\left(d_{i},d_{j}\right)\neq 0,\\ DGS\left(d_{i},d_{j}\right) & \text{ otherwise} \end{array}\right.$$ (6)

In addition, for any given microbe pair *m*_*i*_ and *m*_*j*_, the combined similarity between them is calculated as shown in Eq. 7:

$$MS\left(m_{i},m_{j}\right)=\left\{\begin{array}{cc} \frac{1}{2}\left(MFS\left(m_{i},m_{j}\right)+MGS\left(m_{i},m_{j}\right)\right) & \text{if }\;MFS\left(m_{i},m_{j}\right)\neq 0,\\ MGS\left(m_{i},m_{j}\right) & \text{otherwise} \end{array}\right.$$ (7)

Then, the heterogeneous network $H\in R^{\left(N_{d}+N_{m}\right)\times \left(N_{d}+N_{m}\right)}$ , shown in Eq. 8, can be constructed by combining the above integrated microbe similarity network $DS\in R^{N_{d}\times N_{d}}$ , the integrated disease similarity network $MS\in R^{N_{m}\times N_{m}}$ and the known drug-microbe association network $A\in R^{N_{d}\times N_{m}}$ .

$$H=\left[\begin{array}{cc} DS & A\\ A^{T} & MS \end{array}\right]$$ (8)

Next, the model uses above newly constructed heterogeneous network **H** as an input to the GNN-based encoder to learn the low dimensional embedding representations of the drugs and microbes.

## 4 Methods

**FIGURE 1:**
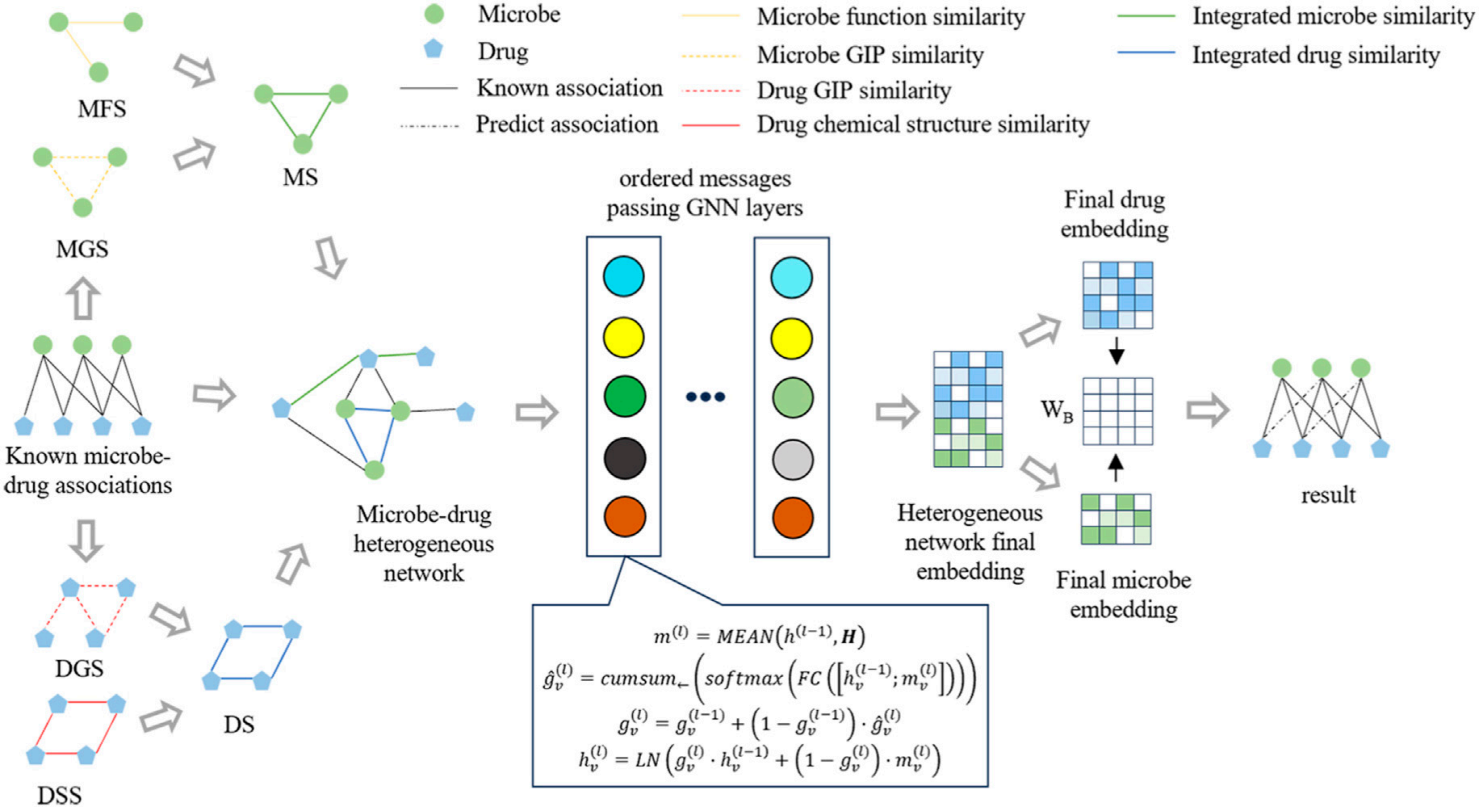
Flowchart of the OGNNMDA.

Figure 1 illustrates the framework of OGNNMDA, comprising three primary modules: the input module, encoder module, and decoder module. The input module is responsible for extracting multiple biomedical information features to be utilized as inputs for OGNNMDA. The encoder module focuses on learning the node embedding representation of the microbes and drugs. Lastly, the decoder module employs bilinear decoders to predict new drug-microbe associations.

### 4.1 Encoder

OGNNMDA is a graph neural network that directly processes the graph as input, effectively utilizing both node information and structural characteristics. Graph neural networks have gained significant popularity in link prediction tasks (Zhang and Chen, 2018), showcasing their widespread adoption. By leveraging the adjacency matrix **H** obtained earlier, Eq. 9 defines the specific formulation of the GNN.

$$h_{v}^{\left(l\right)}=\gamma \left(h_{v}^{\left(l-1\right)},{□}_{u\in N\left(v\right)},\phi \left(h_{v}^{\left(l-1\right)},h_{u}^{\left(l-1\right)},H\right)\right)$$ (9)

Here, $l\in \left\{1\ldots L_{\mathrm{conv}}\right\}$ , $h_{v}^{\left(l\right)}\in R^{1\times k}$ is the embedding feature of the layer *l*, $N\left(v\right)$ denotes the set of neighboring nodes for the node *v*, *L*_*conv*_ corresponds to the number of layers in the GNN network and the number of message-passing rounds. The dimension of the node’s embedding feature is denoted by *k*. In this study, the final embedding dimension is set to match the embedding dimensions used across the GNN layers. **H** is the microbe-drug heterogeneous network graph defined in Eq. 8, which is processed for embedding and provides edge information for the GNN. The node representation $h^{\left(0\right)}\in R^{\left(N_{d}+N_{m}\right)\times k}$ is obtained by a two-layer MLP defined by Eq. 10 and 11. The trainable variables $W_{fc}^{\left(1\right)},W_{fc}^{\left(2\right)}\in R^{\left(N_{d}+N_{m}\right)\times k}$ and $B_{fc}^{\left(1\right)},B_{fc}^{\left(2\right)}\in R^{k}$ are involved in this process. Additionally, $H_{\mathrm{init}}\in R^{\left(N_{d}+N_{m}\right)\times \left(N_{d}+N_{m}\right)}$ represents the initial node representation, and *σ* denotes the ReLU activation function.

$$h^{\left(0\right)}=\sigma \left(W_{fc}^{\left(2\right)}\sigma \left(W_{fc}^{\left(1\right)}H_{\mathrm{init}}+B_{fc}^{\left(1\right)}\right)+B_{fc}^{\left(2\right)}\right)$$ (10)

$$H_{\mathrm{init}}=\left[\begin{array}{cc} 0 & A\\ A^{T} & 0 \end{array}\right]$$ (11)

The function *ϕ* calculates the messages transmitted between nodes, where the edge attribute is directly used as the message. The symbol □ represents the message aggregation function, and in this paper, the mean method is employed (Huan et al., 2021). This means that messages received from multiple neighboring nodes are aggregated by taking their average, resulting in message characteristics used for updating node representations. Finally, *γ* represents the node representation update function, which implements the ordered message-passing mechanism discussed in this paper.

**FIGURE 2:**
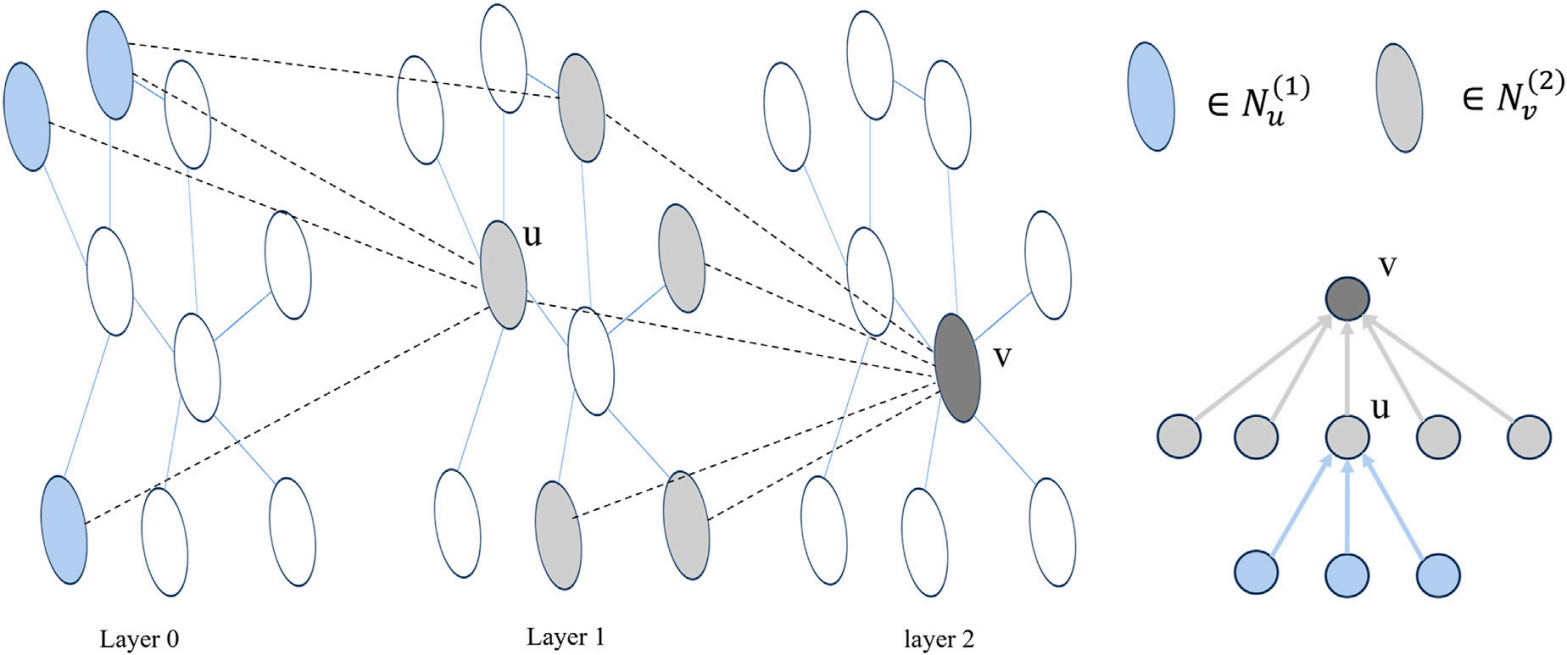
Taking a two-layer GNN as an example, layer 0 represents the initial node embedding, and the adjacency of nodes between layers forms multiple trees. In the figure, *u* is a neighbor node of *v*. $N_{v}^{\left(2\right)}$ and $N_{u}^{\left(1\right)}$ are shown in the image with two colors respectively. The right side shows the tree structure of neighbor information with *v* node as the viewpoint, and the arrow represents the direction of neighbor information transfer.

In the message-passing process of a single-level GNN, a node only exchanges messages with its immediate neighbors. This pattern of neighbor message transmission at different orders aligns with the structure of the node root tree in a multi-layer GNN (Liu et al., 2020). As illustrated in Figure 2, for a node *v*, $N_{v}^{\left(l\right)}$ represents the neighbor information of node *v* at the *l*th layer, and the nesting relationship of its neighbor messages at each layer can be described using Eq. 12.

$$N_{v}^{\left(1\right)}\subseteq N_{v}^{\left(2\right)}\subseteq \cdots \subseteq N_{v}^{\left(L_{\mathrm{conv}}\right)}$$ (12)

In single-layer message passing, direct-neighbor node messages and higher-order neighbor node messages are differentially encoded to ensure orderly message delivery. Specifically, the neuron rows are aligned with the node root tree at each layer, enabling the acquisition of node feature representations with consistent nesting relationships. To implement this alignment encoding method, the neurons can be ordered by linearly arranging the neurons of each layer and considering a segmentation point, denoted as *s*. The information of the neighbors of the current node *v*, at order one or higher, can be encoded as $s_{v}^{\left(l\right)}$ (Song et al., 2023). The segmentation point *s* corresponds to the nested nature of node *v*, and its size relationship is determined by Eq. 13.

$$s_{v}^{\left(1\right)}\leq s_{v}^{\left(2\right)}\leq \cdots \leq s_{v}^{\left(L_{\mathrm{conv}}\right)}$$ (13)

Next, we describe the node feature update function *γ*, which is exemplified below for a specific node *v*. The function can be divided into three distinct steps.

1. Compute the aggregated message representation $m^{\left(l\right)}\in R^{\left(N_{d}+N_{m}\right)\times k}$ for layer *l*.

$$m^{\left(l\right)}=MEAN\left(h^{\left(l-1\right)},H\right)$$ (14)

2. For node *v*, this paper utilizes the gating vector ${\hat{g}}_{v}^{\left(l\right)}$ of dimension $\left(N_{d}+N_{m}\right)$ to describe the segmentation point $s_{v}^{\left(l\right)}$ . Specifically, the value of the left part $\left[0,s_{v}^{\left(l\right)}-1\right]$ is set to 1, indicating the neighboring neurons of node v that are higher than the first order. Conversely, the value of the right part $\left[s_{v}^{\left(l\right)},N_{d}+N_{m}-1\right]$ is set to 0, denoting direct neighboring neurons. This is achieved by calculating the cumulative sum of the probability that each position in the node servers as a split point $s_{v}^{\left(l\right)}$ . The expectation gating vector ${\hat{g}}_{v}^{\left(l\right)}$ is obtained through a biased linear projection of the node representation vector in layer *l* − 1 and the message vector in layer *l*, as shown in Eq. 15.

$${\hat{g}}_{v}^{\left(l\right)}={cumsum}_{\leftarrow }\left(softmax\left(\left[h_{v}^{\left(l-1\right)};m_{v}^{\left(l\right)}\right]W_{g}^{\left(l\right)}+B_{g}^{\left(l\right)}\right)\right)$$ (15)

In Eq. 15, the trainable parameters $W_{g}^{\left(l\right)}\in R^{2k\times k}$ and $B_{g}^{\left(l\right)}\in R^{k}$ are utilized. Additionally, $\left[h_{v}^{\left(l-1\right)};m_{v}^{\left(l\right)}\right]$ represents the concatenation of two vectors $h_{v}^{\left(l-1\right)}$ and $m_{v}^{\left(l\right)}$ . To ensure that the predicted gated vector ${\hat{g}}_{v}^{\left(l\right)}$ adheres to the relative size relationship of the splitting points mentioned earlier, the operation described in Eq. 16. This operation yields the final gated vector $g_{v}^{\left(l\right)}$ .

$$g_{v}^{\left(l\right)}=g_{v}^{\left(l-1\right)}+\left(1-g_{v}^{\left(l-1\right)}\right)\cdot {\hat{g}}_{v}^{\left(l\right)}$$ (16)

3. Equation 17 demonstrates the utilization of the gating vector $g_{v}^{\left(l\right)}$ to regulate the integration of the layer *l* − 1 node representation $h_{v}^{\left(l-1\right)}$ with the layer *l* aggregated context $m_{v}^{\left(l\right)}$ . This process results in the acquisition of the new node representation $h_{v}^{\left(l\right)}$ .

$$h_{v}^{\left(l\right)}=LN\left(g_{v}^{\left(l\right)}\cdot h_{v}^{\left(l-1\right)}+\left(1-g_{v}^{\left(l\right)}\right)\cdot m_{v}^{\left(l\right)}\right)$$ (17)

In Eq. 17, the symbol ⋅ represents element-by-element multiplication, and LN refers to the layer normalization operation (Chen et al., 2022).

### 4.2 Decoder

After the previous rounds of the ordered message passing process, the final node embedding representation $h^{\left(L_{\mathrm{conv}}\right)}\in R^{\left(N_{d}+N_{m}\right)\times k}$ is obtained. This representation can be considered as the concatenation of the final embedding features of the drugs, $h_{d}\in R^{N_{d}\times k}$ , and the microbes, $h_{m}\in R^{N_{m}\times k}$ . In this paper, the final embedding features *h*_*d*_ and *h*_*m*_ are obtained separately using the matrix splicing approach defined in Eq. 18.

$$\left[\begin{array}{c} h_{d}\\ h_{m} \end{array}\right]=h^{\left(L_{\mathrm{conv}}\right)}$$ (18)

To reconstruct the adjacency matrix **A**′ representing possible microbe-disease associations, the bilinear decoder is employed. It is a structural component employed for predicting the probability of potential edges or links based on node embedding vectors. These decoders commonly integrate the embedding vectors of node pairs within a graph to generate a score function that assesses the likelihood of a link between two nodes. The key characteristic of bilinear decoders lies in their utilization of bilinear transformations to capture the interaction effects among nodes. Specifically, for a drug node and microbe node pair (u, v) with their respective embedding vectors **h**_*d*_(*u*) and **h**_*m*_(*v*), a bilinear decoder might compute the score by Eq. 19.

$$score\left(h_{d}\left(u\right),h_{m}\left(v\right)\right)={h_{d}\left(u\right)}^{T}Wh_{m}\left(v\right)$$ (19)

Where *W* is a learnable weight matrix. This score can be interpreted as the probability of link occurrence after a nonlinear activation function transformation, so that **A**′ can be obtained by the bilinear decoder as shown in Eq. 20.

$$A^{\prime }=\sigma \left(h_{d}W_{B}h_{m}^{T}\right)$$ (20)

In the above formula, where $W_{B}\in R^{k\times k}$ represents a trainable matrix and $\sigma \left(x\right)=1/\left(1+e^{-x}\right)$ is the sigmoid function. Overall, the complete computational flow of OGNNMDA can be seen in Algorithm 1.

Algorithm 1 OGNNMDA.

**Require:** Known associations matrix $A\in R^{N_{d}\times N_{m}}$ , drug similarity matrix $DS\in R^{N_{d}\times N_{d}}$ , microbe similarity matrix $MS\in R^{N_{m}\times N_{m}}$ and *α* = 600 is the number of iterations for OGNNMDA
**Ensure:** The constructed drug-microbe associations matrix $A^{\prime }\in R^{N_{d}\times N_{m}}$

  1:  Construct the heterogeneous network *H* according to formula (8)
  2:  Initialize the embedding feature matrix *H*_*init*_ according to formula (11).
  3:  Initialize the gate vector = 0
  4:  **for** *i* = 1 → *α* **do**
  5:   calculate **h**_0_ according to formula (10)
  6:   **for** *l* = 1 → *L*_*conv*_ **do**
  7:    calculate message matrix $m^{\left(l\right)}$ formula (14).
  8:    calculate ${\hat{g}}^{\left(l\right)}$ by formula (15)
  9:    calculate ${\tilde{g}}^{\left(l\right)}$ formula (16)
  10:    calculate $h^{\left(l\right)}$ formula (17)
  11:   **end for**
  12:   get the embedding feature for drugs and microbes with *h*_*d*_ and *h*_*m*_ according to formula (18)
  13:   get the reconstruction matrix *A*′ by formula (20)
  14:  **end for**

### 4.3 Optimization

During the experiment, positive samples were the drug-microbe pairs with known associations, while negative samples were the drug-microbe pairs without known associations. These sets of positive and negative samples are denoted as Ω^+^ and Ω^−^, respectively, for ease of description. It is important to note that the number of pairs with known associations in both the aBiofilm dataset and the MDAD dataset is significantly smaller than the number of pairs without known associations. Therefore, when training OGNNMDA, the loss function incorporates a weighted cross-entropy loss, as defined in Eq. 21.

$$L=-\frac{1}{N_{d}\times N_{m}}\left(\lambda \underset{\left(i,j\right)\in \Omega^{+}}{\sum }\log\left(a_{i,j}^{\prime }\right)+\underset{\left(i,j\right)\in \Omega^{-}}{\sum }\log\left(1-a_{i,j}^{\prime }\right)\right)$$ (21)

In the above formula, (*i*, *j*) represents a pair of the drug *d*_*i*_ and microbe *m*_*j*_. *λ* is introduced as a balancing factor, calculated as the ratio of the number of samples in Ω^−^ to the number of samples in Ω^+^. This factor helps attenuate the impact of data imbalance and emphasizes the reinforcement of known correlation information.

In this paper, the Xavier initialization method (Duong et al., 2019) is employed to initialize the trainable parameter matrices in various components of the model. These include the 2-layer fully connected layer, the ordered message-passing graph neural network layer, the bilinear decoder, and others, denoted as $\left\{W_{fc}^{\left(l\right)},B_{fc}^{\left(l\right)}|W_{fc}^{\left(l\right)}\in R^{\left(N_{d}+N_{m}\right)\times k},B_{fc}^{\left(l\right)}\in R^{k},1\leq l\leq K_{fc}\right\}$ , $\left\{W_{g}^{\left(l\right)},B_{g}^{\left(l\right)}|W_{g}^{\left(l\right)}\in R^{\left(2*k\right)\times cs},B_{g}^{\left(l\right)}\in R^{cs},1\leq l\leq K_{\mathrm{conv}}\right\}$ , and the bias matrix $W_{B}\in R^{k\times k}$ . Furthermore, the Adam optimizer (Wang et al., 2023) is utilized to minimize the loss function. Adam combines the benefits of momentum optimization and adaptive learning rate, enabling quick convergence and adaptation to different parameter learning rates during the training process. This optimization technique enhances the training effectiveness of the deep learning model.

To prevent overfitting, the paper introduces node dropout (Piotrowski et al., 2020) and regularized dropout (Berg et al., 2017) schemes in the graph convolution layer. Node dropout can be seen as training multiple models on various sub-nodes, and the combination of these sub-nodes is used to predict unknown microbe-drug pairs (Tan et al., 2020).

## 5 Results

This paper begins by providing a brief overview of the experimental setup and the analysis and selection of certain hyperparameters. The aim is to validate the predictive performance advantages of OGNNMDA through intensive comparison experiments. These experiments involve 6 representative microbe-drug association prediction models, including state-of-the-art approaches. The evaluation is conducted on three well-known public datasets, namely, aBiofilm, MDAD and DrugVirus, within a 5-fold cross-validation framework. Furthermore, ablation experiments are performed to investigate the effectiveness of the ordered message-passing mechanism employed in OGNNMDA. Finally, a case study is presented to validate OGNNMDA using two commonly used drugs, ciprofloxacin and moxifloxacin, along with two common oral microbes, Actinobacillus aggregatum and *Clostridium* nucleatum.

### 5.1 Experimental parameter setting

In this paper, all experimental evaluations are conducted within a five-fold cross-validation setup. To ensure statistical robustness, each method is executed ten independent times for every experiment, thereby enabling the calculation of the mean value for each performance metric across these repetitions. In detail, this involves dividing all known associations in the dataset equally into 5 parts, denoted as $test_{p}=\left\{tp_{1},tp_{2},tp_{3},tp_{4},tp_{5}\right\}$ . Additionally, a subset of the same size as the known associations is randomly selected from the unknown association set. This subset is divided equally into 5 parts, denoted as $test_{n}=\left\{tn_{1},tn_{2},tn_{3},tn_{4},tn_{5}\right\}$ .

During the *i* − *th* (1 ≤ *i* ≤ 5) cross-validation iteration, the training set is defined as $train_{i}=test_{p}-\left\{tp_{i}\right\}$ , and the test set is defined as $test_{i}=\left\{tp_{i}\right\}\cup \left\{tn_{i}\right\}$ . The final test result of the 5-fold cross-validation experiment is calculated based on the combined test set, *test* = *test*_*p*_ ∪ *test*_*n*_.

Based on the previous description of the model structure, OGNNMDA incorporates several hyperparameters, including the dimension size (*k*) of embedded features, the number of fully-connected layers (*L*_*fc*_), the number of ordered message-passing GNN layers (*L*_*conv*_), the initial learning rate (*r*) of Adam’s optimizer, the total training period (*α*), the node dropout metrics (*β*), and the regularized dropout parameter (*γ*).

To establish initial values for these parameters, we set *L*_*fc*_ = 2, *r* = 0.008, *α* = 600, *β* = 0.6, and *γ* = 0.4. Subsequently, we examine the effects of different values for parameters *k* and *L*_*conv*_ through experimental analysis.

**FIGURE 3:**
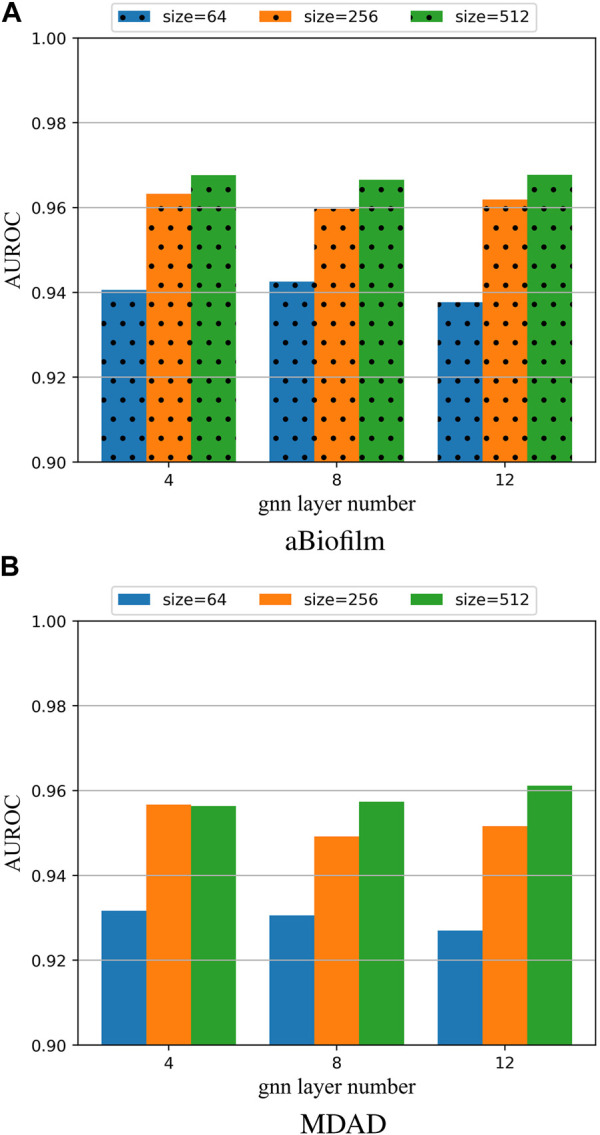
**(A)** Model hyperparameter analysis on the aBiofilm dataset. **(B)** Model hyperparameter analysis on the MDAD dataset.

To investigate the impact of different hyperparameter values on the model, this paper performed 5-fold cross-validation (5 cv) experiments on the aBiofilm and MDAD datasets. The results for the AUROC were plotted in Figure 3, showcasing the outcomes for various combinations of the parameters *L*_*conv*_ and *k*.

From Figures 3A, B, it is evident that the optimal combination of *L*_*conv*_ and *k* is *L*_*conv*_ = 12 and *k* = 512. Therefore, this parameter setting will be utilized for OGNNMDA in subsequent experiments.

### 5.2 Comparison experiments

In this study, we replicate the code and data based on publicly accessible resources of these six methodologies, with all competing methods’ parameter configurations set according to their optimal values as reported in their respective publications. The 6 methods we compared OGNNMDA with are HMDAKATZ (Zhu et al., 2019a), GCNNMDA (Long et al., 2020), GSAMDA (Tan et al., 2022), SCSMDA (Tian et al., 2023), LAGCN (Yu et al., 2021), and NTSHMDA (Luo and Long, 2018), which are widely used in linkage prediction problems across various bioinformatics domains. However, due to GSAMDA not having performed experiments on DrugVirus in their paper nor specifying the construction process for the microbe-disease associations and drug-disease associations used to derive disease-based microbial and drug-Hamming similarities, comparative evaluations on DrugVirus are limited to the remaining five competing approaches.

**FIGURE 4:**
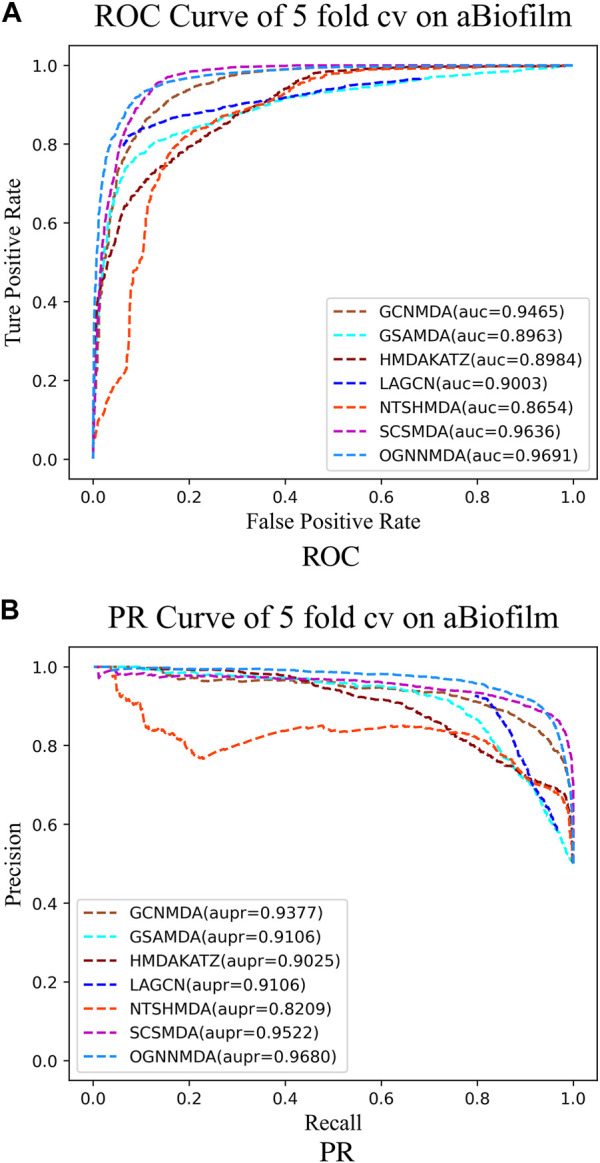
**(A)** ROC curves for each modeling approach based on the aBiofilm dataset 5-cv. **(B)** PR curves for each modeling approach based on the aBiofilm dataset 5-cv.

**FIGURE 5:**
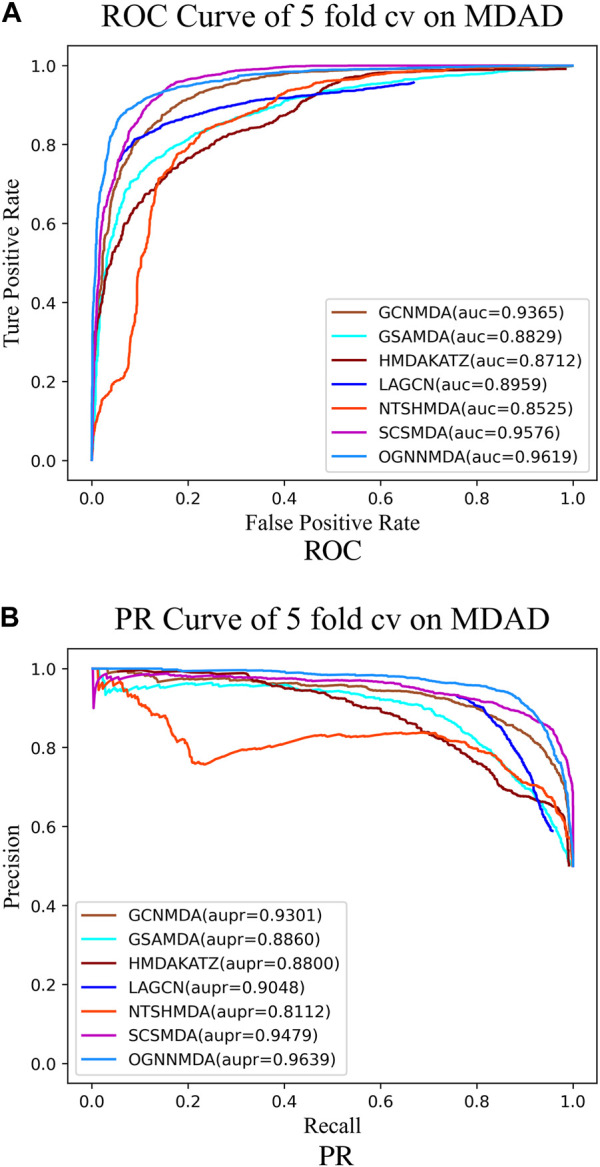
**(A)** ROC curves for each modeling approach based on the MDAD dataset 5-cv. **(B)** PR curves for each modeling approach based on the MDAD dataset 5-cv.

**FIGURE 6:**
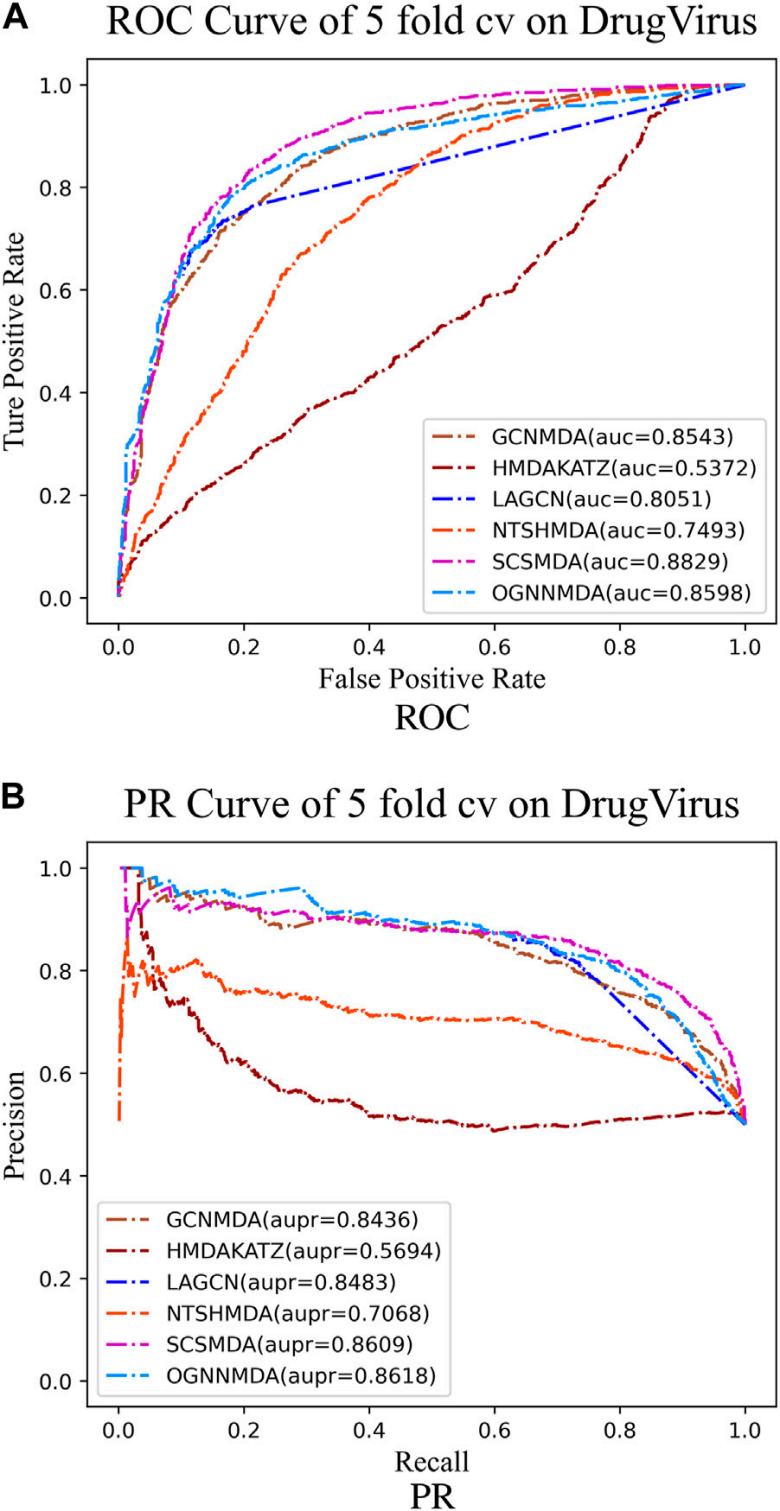
**(A)** ROC curves for each modeling approach based on the DrugVirus dataset 5-cv. **(B)** PR curves for each modeling approach based on the DrugVirus dataset 5-cv.

To train and evaluate these methods, a 5-fold cross-validation experimental framework was employed. Performance evaluation was based on metrics such as AUC, AUPR, accuracy, and F1 score, chosen for their effectiveness in assessing performance. The experimental results, including the performance metrics, are presented in Tables 2–4. Additionally, ROC curves (see Figure 4A, 5A, 6A) and PR curves (see Figure 4B, 5B, 6B) were plotted to facilitate comparison among the different methods on the respective datasets.

**TABLE 2:** Comparison of AUC, AUPR, Acc, and F1-score obtained by each method based on aBiofilm dataset at 5-cv.

Methods	AUC	AUPR	Accuracy	F1-score
GCNMDA	0.9465 ± 0.0001	0.9376 ± 0.0001	0.8772 ± 0.0004	0.8819 ± 0.0002
GSAMDA	0.8955 ± 0.0051	0.9073 ± 0.0053	0.8345 ± 0.0058	0.8295 ± 0.0055
HMDAKATZ	0.8982 ± 0.0027	0.9018 ± 0.0026	0.7811 ± 0.0112	0.8088 ± 0.0040
LAGCN	0.8991 ± 0.0032	0.9084 ± 0.0030	0.8710 ± 0.0032	0.8651 ± 0.0036
NTSHMDA	0.8633 ± 0.0050	0.8204 ± 0.0076	0.8073 ± 0.0082	0.8117 ± 0.0045
SCSMDA	0.9628±0.0021	0.9504±0.0035	0.9083±0.0038	0.9121±0.0035
OGNNMDA	***0.9693 ± 0.0008***	***0.9690 ± 0.0009***	***0.9141 ± 0.0031***	***0.9152 ± 0.0026***

Bold values are the best performing of all these comparison methods, and the next best values are underlined.

**TABLE 3:** Comparison of AUC, AUPR, Acc and F1-score obtained by each method based on MDAD dataset at 5-cv.

Methods	AUC	AUPR	Accuracy	F1-score
GCNMDA	0.9365 ± 0.0001	0.9300 ± 0.0002	0.8617 ± 0.0011	0.8675 ± 0.0004
GSAMDA	0.8760 ± 0.0197	0.8823 ± 0.0164	0.7979 ± 0.0279	0.8028 ± 0.0176
HMDAKATZ	0.8717 ± 0.0039	0.8798 ± 0.0045	0.7691 ± 0.0167	0.7856 ± 0.0046
LAGCN	0.8974 ± 0.0056	0.9062 ± 0.0050	0.8572 ± 0.0067	0.8536 ± 0.0061
NTSHMDA	0.8512 ± 0.0043	0.8094 ± 0.0055	0.7820 ± 0.0137	0.8028 ± 0.0044
SCSMDA	0.9574±0.0022	0.9478±0.0036	0.8953±0.0045	0.8996±0.0038
OGNNMDA	***0.9616 ± 0.0021***	***0.9645 ± 0.0024***	***0.9048 ± 0.0032***	***0.9047 ± 0.0026***

Bold values are the best performing of all these comparison methods, and the next best values are underlined.

**TABLE 4:** Comparison of AUC, AUPR, Acc and F1-score obtained by each method based on DrugVirus dataset at 5-cv.

Methods	AUC	AUPR	Accuracy	F1-score
GCNMDA	0.8541 ± 0.0004	0.8441 ± 0.0006	0.7732 ± 0.0045	0.7912 ± 0.0007
HMDAKATZ	0.5356 ± 0.0080	0.5669 ± 0.0057	0.5397 ± 0.0054	0.6835 ± 0.0022
LAGCN	0.8044 ± 0.0079	0.8460 ± 0.0076	0.7794 ± 0.0067	0.7744 ± 0.0055
NTSHMDA	0.7482 ± 0.0087	0.7039 ± 0.0092	0.6789 ± 0.0130	0.7395 ± 0.0070
SCSMDA	***0.8810 ± 0.0053***	0.8590±0.0102	***0.8098 ± 0.0071***	***0.8201 ± 0.0038***
OGNNMDA	0.8591±0.0076	***0.8633 ± 0.0078***	0.7916±0.0115	0.7990±0.0077

Bold values are the best performing of all these comparison methods, and the next best values are underlined.

Based on the experimental results from Table 2, it is evident that OGNNMDA achieves the highest AUC values on the aBiofilm dataset, with an average AUC of 0.9693 ± 0.0008. This is 0.65% higher than the next highest AUC value of 0.9628 ± 0.0021 obtained by SCSMDA. OGNNMDA also outperforms other methods in terms of AUPR, Accuracy, and F1-Score, with values of 0.9690 ± 0.0009, 0.9141 ± 0.0031, and 0.9151 ± 0.0026, respectively.

Similarly, in Table 3, which presents the results on the MDAD dataset, OGNNMDA exhibits superior performance across all four evaluation metrics. The comparison between the two tables suggests that OGNNMDA performs better on the aBiofilm dataset compared to MDAD. This disparity can be attributed to the sparser nature of the data in MDAD, resulting in a smaller ratio of positive to negative samples and a more pronounced sample imbalance issue.

Finally, we examine the results from Table 4, which presents the performance of all methods on the DrugVirus dataset. OGNNMDA achieved the highest AUPR score with a mean value of 0.8633 ± 0.0078; however, SCS-MDA outperformed others in terms of the AUC (0.8810 ± 0.0053), Accuracy (0.8098 ± 0.0071), and F1-score (0.8201 ± 0.0038). Notably, OGNNMDA did not maintain its leading position on the DrugVirus dataset as it did on the aBiofilm and MDAD datasets. This relative underperformance may be attributed to the smaller scale of the DrugVirus dataset compared to aBiofilm and MDAD, potentially limiting OGNNMDA’s ability to effectively train its more complex weighting parameters for optimal prediction.

### 5.3 Ablation experiment

To evaluate the efficacy of the ordered message-passing mechanism, this section presents ablation experiments, the results of which are presented in Table 5. In this context, GNN refers to a simple graph neural network model utilizing a mean aggregator as an encoder, while OGNN represents an enhanced ordered message-passing graph neural network model based on GNN, specifically the model proposed in this paper, OGNNMDA. The evaluation entails 5-fold cross-validation experiments on the aBiofilm and MDAD datasets, with specific parameter settings described in previous sections.

**TABLE 5:** Results of ablation experiments.

Dataset	Method	AUC	AUPR	Accuracy	F1-score
aBiofilm	GNN	0.8940 ± 0.0025	0.9090 ± 0.0040	0.8359 ± 0.0036	0.8337 ± 0.0033
aBiofilm	OGNN	***0.9673 ± 0.0014***	***0.9681 ± 0.0021***	***0.9111 ± 0.0025***	***0.9119 ± 0.0024***
MDAD	GNN	0.8872 ± 0.0026	0.9027 ± 0.0037	0.8333 ± 0.0043	0.8334 ± 0.0035
MDAD	OGNN	***0.9595 ± 0.0020***	***0.9616 ± 0.0022***	***0.9014 ± 0.0025***	***0.9013 ± 0.0027***

Bold values are the best performing on the same dataset.

Based on the data presented in Table 5, the underlying GNN encoder exhibits poor performance on both datasets, showing a significant gap in all metrics compared to the OGNNMDA model utilizing OGNN as the encoder. Therefore, it is reasonable to conclude that the ordered message-passing mechanism effectively enhances the embedding performance of GNN, leading to improved prediction results in microbe drug association prediction.

### 5.4 Case study

To validate the prediction performance of OGNNMDA, case study experiments were conducted using two popular drugs and two microbes as targets. First, OGNNMDA was trained on the complete aBiofilm dataset to obtain the predicted association information neighbor matrix. Then, the top 20 most relevant objects for each target microbe and drug were filtered out. Finally, the relevant published PubMed literature was searched to validate the predicted microbe-drug association pairs against existing references. The first drug selected for the case study was ciprofloxacin, a fluorinated quinolone antibiotic, which has been extensively studied and shown to be associated with a wide range of human microbiome (Yayehrad et al., 2022). For instance, Rehman et al. (2019) demonstrated the effectiveness of amphotericin-B and 5% ciprofloxacin in blocking the growth mechanisms of *Pseudomonas aeruginosa* and *Candida* albicans. Ciprofloxacin has also shown susceptibility against *Staphylococcus aureus*, *Staphylococcus* epidermidis, *Mycobacterium* subspecies, *Escherichia coli*, and *Mycobacterium tuberculosis* (Smirnova and Oktyabrsky, 2018). The second drug chosen for the case study is moxifloxacin, a fluoroquinolone antibiotic (Rodríguez-López et al., 2020), known to be associated with antibiotic-resistant bacteria (ARB) (Loyola-Rodriguez et al., 2018) and *Listeria* monocytogenes (Rodríguez-López et al., 2020). The specific experimental results for the two drugs are presented in Tables 6, 7, respectively. These tables provide supporting literature information for the top 20 predicted microbes associated with ciprofloxacin and moxifloxacin. Upon observing Tables 6, 7, it is evident that 20 and 17 out of the top 20 predicted microbes associated with ciprofloxacin and moxifloxacin, respectively, have been validated by the available literature.

**TABLE 6:** Top 20 related microbes to Ciprofloxacin predicted by OGNNMDA.

Rank	Microbe name	Evidence	Rank	Microbe name	Evidence
1	*Proteus* vulgaris	PMID: 27303616	11	*Candida* albicans	PMID: 35404123
2	*Morganella morganii*	PMID: 25107625	12	Burkholderia thailandensis	PMID: 31404671
3	Providencia stuartii	PMID: 23029216	13	*Serratia marcescens*	PMID: 27085794
4	*Pseudomonas aeruginosa*	PMID: 30605076	14	*Streptococcus* mutans	PMID: 33402618
5	Stenotrophomonas maltophilia	PMID: 30448331	15	*Vibrio cholerae*	PMID: 28270803
6	*Escherichia coli*	PMID: 29228224	16	*Vibrio* harveyi	PMID: 32019500
7	*Staphylococcus aureus*	PMID: 36499677	17	*Pseudomonas* putida	PMID: 19280293
8	Burkholderia pseudomallei	PMID: 27936915	18	*Bacillus subtilis*	PMID: 33218776
9	*Klebsiella pneumoniae*	PMID: 28223459	19	*Staphylococcus* epidermidis	PMID: 9111541
10	*Proteus mirabilis*	PMID: 27303616	20	Burkholderia cenocepacia	PMID: 34116184

**TABLE 7:** Top 20 related microbes to Moxifloxacin predicted by OGNNMDA.

Rank	Microbe name	Evidence	Rank	Microbe name	Evidence
1	*Candida* albicans	PMID: 12121916	11	*Streptococcus* mutans	PMID: 29392681
2	Stenotrophomonas maltophilia	PMID: 31748318	12	*Candida* dubliniensis	PMID: 30237975
3	*Pseudomonas aeruginosa*	PMID: 31643179	13	*Candida* parapsilosis	PMID: 20455400
4	*Mycobacterium avium*	PMID: 31239192	14	Mixed Culture of bacteria and fungus	PMID: 31732485
5	*Candida* glabrata	PMID: 30768071	15	*Staphylococcus* epidermidis	PMID: 35214102
6	*Staphylococcus aureus*	PMID: 33512346	16	Eikenella corrodens	PMID: 35023367
7	*Candida tropicalis*	PMID: 20455400	17	*Escherichia coli*	PMID: 36250047
8	Burkholderia multivorans	Unconfirmed	18	Burkholderia thailandensis	Unconfirmed
9	Burkholderia cenocepacia	PMID: 33120688	19	*Candida* guiliermondi	Unconfirmed
10	*Candida* krusei	PMID: 22993935	20	*Acinetobacter* baumannii	PMID: 12951327

Furthermore, the first microbe selected for the case study was Aggregate Actinobacteria Accompanying Bacteria, a Gram-negative bacterium belonging to the family Pasteuriaceae (Krueger and Brown, 2020). It is primarily found in the oral cavity and is associated with various oral diseases and systemic infections (Jensen et al., 2019). In terms of its impact on human health, aggregates of Actinobacillus companionis are commonly linked to periodontal diseases, particularly aggressive forms of periodontitis. This bacterium has the ability to invade and colonize periodontal tissues, leading to inflammation, destruction of the periodontal ligament, and bone loss. Consequently, it is often found at a higher rate in individuals with severe periodontal disease. Sol et al. demonstrated that sub-killer concentrations of LL-37, Cathelicidin, and Scrambled LL-37 inhibit the biofilm formation of Actinobacillus actinomycetemcomitans and act as conditioning agents and lectins, greatly enhancing clearance by neutrophils and macrophages (Sol et al., 2013). Basavaraju et al. found that AHL lactonase hydrolyzes the lactone ring in the high serine portion of AHL, without affecting the rest of the signaling molecular structure. This inhibitory effect of AHL lactonase on group sensing of actinomycete aggregates has been observed (Basavaraju et al., 2016). The second microbe chosen for the case study was *Clostridium* nucleatum, a bacterium known for causing opportunistic infections and recently associated with colorectal cancer (Brennan and Garrett, 2019). In this study, Tables 8, 9 present the top 20 predicted drugs that are most relevant to Aggregate Actinobacteria Accompanying Bacteria and *Clostridium* nucleatum, respectively. Based on the information in the tables, 17 out of the top 20 predicted drugs for Aggregate Actinobacteria Accompanying Bacteria and 18 out of the top 20 predicted drugs for *Clostridium* nucleatum have been validated in the existing literature. Therefore, it can be concluded that OGNNMDA achieves satisfactory predictive performance in both microbe and drug case studies.

**TABLE 8:** Top 20 drugs associated with the microbe Aggregatibacter actinomycetemcomitans predicted by OGNNMDA.

Rank	Drug name	Evidence	Rank	Drug name	Evidence
1	LL-37	PMID: 23836819	11	N-Acetylcysteine	PMID: 18038907
2	Cathelicidin	PMID: 23836819	12	L-Aspartate	PMID: 10769165
3	Hamamelitannin	PMID: 26561076	13	3-(2-Furylmethyl)-2-{[(5-hydroxy-1H-pyrazol-3-yl)methyl]sulfanyl}-3,5,6,7-tetrahydro-4H-cyclopenta [4,5]thieno [2,3-d]pyrimidin-4-one	Unconfirmed
4	Scrambled LL-37	PMID: 23836819	14	Curcumin	PMID: 33065303
5	Culture supernatant of *Bacillus* licheniformis sp. SP1	Unconfirmed	15	SMAP-29	PMID: 26196513
6	Vancomycin	PMID: 31516229	16	Toremifene	PMID: 26426681
7	AHL lactonase	PMID: 30894996	17	Stem extract of Acacia arabica	PMID: 25114940
8	DispersinB-KSL-W wound gel	Unconfirmed	18	Bark extract of Tamarix aphylla L	PMID: 22963838
9	Epigallocatechin Gallate	PMID: 33793838	19	Magainin-I	PMID: 32104827
10	Farnesol	PMID: 32808302	20	Patulin	PMID: 34271147

**TABLE 9:** Top 20 drugs associated with the microbe *Fusobacterium* nucleatum as predicted by OGNNMDA.

Rank	Drug name	Evidence	Rank	Drug name	Evidence
1	Green tea polyphenols	PMID: 28322293	11	Lactoferricin B	PMID: 33249255
2	Bark extract of Tamarix aphylla L	Unconfirmed	12	Vancomycin	PMID: 30349083
3	Stem extract of Acacia arabica	PMID: 25654035	13	Penicillic acid	PMID: 10223950
4	AHL lactonase	PMID: 32555242	14	LL-37	PMID: 21220789
5	Patulin	PMID: 26574491	15	Hamamelitannin	PMID: 27983597
6	L-Aspartate	PMID: 3875311	16	Competence Stimulating Peptide	PMID: 36371909
7	Culture supernatant of *Bacillus* licheniformis sp. SP1	PMID: 22730907	17	Cell-free supernatant of *Pseudomonas* fluorescens	PMID: 36891385
8	Lys-a1	Unconfirmed	18	C6-HSL	PMID: 32555242
9	Curcumin	PMID: 26246690	19	G H12	PMID: 31389653
10	Epigallocatechin Gallate	PMID: 34402021	20	N-Acetylcysteine	PMID: 25568806

## 6 Conclusion and discussion

This paper proposes OGNNMDA, a novel deep learning model for predicting potential microbe-drug associations, based on graph neural networks (GNNs) with an ordered message-passing mechanism. OGNNMDA utilizes multiple sources of biological data to construct similarity features for drugs and microbes, which are combined to form a heterogeneous network containing association and similarity information. To obtain drug and microbe embeddings, a multilayer GNN with ordered message passing is employed to differentiate node neighborhood messages during the message passing stage. A bilinear decoder is then used to generate association prediction scores. The OGNNMDA methodology was subjected to a rigorous evaluation regimen, encompassing comparative experiments on the aBiofilm and MDAD datasets as well as the DrugVirus dataset, where it utilized a 5-fold cross-validation scheme. The empirical outcomes revealed that OGNNMDA surpassed the current state-of-the-art performance benchmarks on both the aBiofilm and MDAD datasets. However, in the context of the DrugVirus dataset, OGNNMDA demonstrated a commendable yet second-best performance compared to existing methods. For clarity, while comprehensive experimental evaluations including comparative analyses were conducted for the DrugVirus dataset, the ablation experiments and case studies were confined to the aBiofilm and MDAD datasets alone. Despite this, the overall results affirm OGNNMDA’s robustness and competitive advantage in predicting potential microbe-drug associations across different datasets. The main contributions of this model can be summarized as follows.

1. It fully leverages additional biomedical data, such as microbe functional similarity based on microbial genomic information and drug molecular structural phase-based feature similarity.
2. It introduces an improved GNN model with an ordered message-passing mechanism, which achieves better embedding performance by distinguishing node neighbor messages.
3. The overall model outperforms existing state-of-the-art methods for predicting potential microbe-drug associations.

However, OGNNMDA is not without its limitations. The model’s performance is contingent upon the scale of the accessible dataset; with a relatively modest-sized corpus, the inherent sparsity in the microbial-drug association adjacency matrix can potentially impede the exhaustive exploitation of the graph’s structural information and limit the expressiveness of the learned embeddings. Furthermore, OGNNMDA homogenously handles microbial and drug nodes within the network without explicitly accounting for their distinctive patterns of interaction. In light of these challenges, future research directions can be directed towards:

1. Expanding Feature Representation: Augmenting the existing feature space by integrating supplementary biomedical data such as genomic sequences of microbes (Deng et al., 2022) and pharmacological similarity based on side effect profiles (Zheng et al., 2019). This enrichment could provide deeper insights into the intrinsic properties of both microorganisms and drugs, thereby enhancing the quality of the representations learned.
2. Addressing Sparsity Issues: Investigating innovative techniques to tackle the issue of sparse associations, which might involve adopting advanced link prediction strategies or devising specialized regularization methods that are tailored for sparse graphs. These approaches could ensure more efficient utilization of available relational information.
3. Adaptation of Graph Contrastive Learning: Exploring the potential benefits of incorporating graph contrastive learning (GCL) paradigms to improve the robustness and generalizability of the learned embeddings. GCL has shown promise in other domains by extracting meaningful node or graph representations from limited or unlabeled data, hence it could be a viable avenue to mitigate the impact of small datasets on OGNNMDA’s performance (Cai et al., 2023).
4. Refinement of Message-Passing Mechanisms: Examining alternative graph neural network architectures like Graph Attention Networks (GATs) and Graph Convolutional Networks (GCNs), and refining their message-passing processes to better suit the unique characteristics of the microbial-drug association problem.

By systematically addressing these limitations and venturing into new methodological frontiers, future iterations of OGNNMDA and similar models are poised to achieve heightened accuracy and resilience in predicting microbe-drug associations, thus contributing significantly to this burgeoning research domain.

## Data Availability

The original contributions presented in the study are included in the article/Supplementary material, further inquiries can be directed to the corresponding author.
